# 2213. History of Infection and Worry about COVID-19 Drive Antibiotic Use for “Flu- or COVID-like” Symptoms in the United States and Mexico during the Pandemic

**DOI:** 10.1093/ofid/ofad500.1835

**Published:** 2023-11-27

**Authors:** Jose Luis Camarena, Brooke Hawkes, Edward Bedrick, Katherine Ellingson

**Affiliations:** University of Arizona College of Public Health, Tucson, Arizona; University of Arizona College of Public Health, Tucson, Arizona; University of Arizona, Tucson, Arizona; University of Arizona, Tucson, Arizona

## Abstract

**Background:**

Antibiotic resistance has worsened since the start of the COVID-19 pandemic because of overstrained healthcare and laboratory systems, disrupted antimicrobial stewardship programs, and overuse of antibiotics. Community-based antibiotic use during the pandemic in the United States (US) and Mexico, including the socially vulnerable border region, is poorly characterized and does not account for non-prescription antibiotic use. The goal of this study was to assess the association between antibiotic use and infection with, or worry about, COVID-19 in the US and Mexico.

Results for Univariate and Multiple Logistic Regression Analysis for Antibiotic Use with Worry about COVID-19 (n=618) as a Predictor during the COVID-19 Pandemic.

**Figure:**
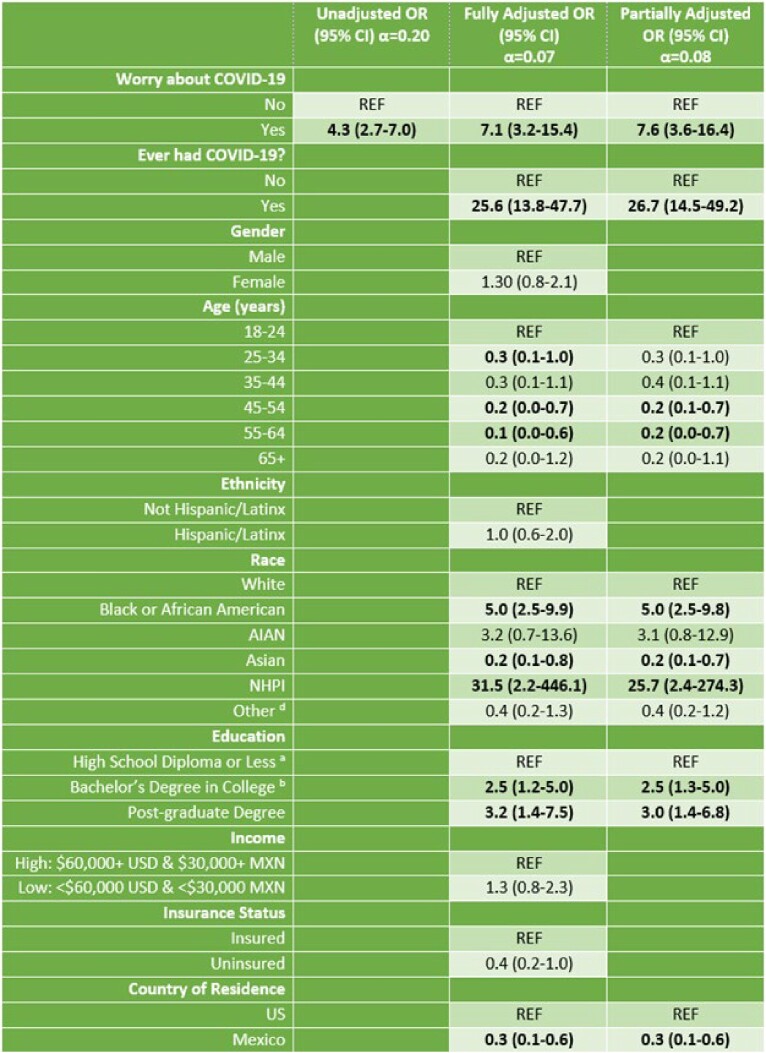

a=High school diploma or equivalent including GED, b=4-year, and c=Household income in previous year before taxes, d=Other includes multiple races, e= adjusted for only significant covariates: COVID-19 infection status, age, race, education, and country of residence. Acronyms: AIN=American Indian or Alaskan Native, MXN= Mexican peso, NHPI=Native Hawaiian or Pacific Islander, OR= odds ratio, SD=standard deviation, USD= US Dollars. Significant findings in bold.

**Methods:**

An online cross-sectional survey with monetary incentive was deployed through Amazon Mechanical Turk to adults in the US and Mexico with enhanced recruitment through flyers at clinics and COVID-19 testing sites in the border regions of the US and Mexico (defined as within 100km of the border). Responses (n=981) were collected from 8/8/2020 to 8/4/2021. Logistic regression models were specified to model any (prescription and non-prescription) antibiotic use for “flu- or COVID-like” symptoms since March 2020. Predictors of interest were history of laboratory-confirmed SARS-CoV-2 infection and general worry about COVID-19. Models were adjusted for sex, age, ethnicity, race, education, income, insurance status, and country of residence.

**Results:**

A total of 688 participants were used in the analysis, excluding 293 who did not answer the antibiotic use question. 29% reported already being infected and 78% reported being worried about COVID-19. After adjusting for significant covariates, those who reported a history of infection and worries about COVID-19 at the time of the survey had increased odds of using antibiotics for flu- or COVID-like symptoms: OR=26.7 (95% CI: 12.5-49.2) and OR=7.6 (95% CI: 3.6-16.4), respectively. Conversely, residing in Mexico decreased the odds of antibiotic use for those who had concerns about COVID-19: OR=0.3 (95% CI: 0.1-0.6) in adjusted models.

**Conclusion:**

Findings suggest that worry about COVID-19 drove higher odds of antibiotic use, which could potentially be attributed to uncertainty, misinformation, and political turmoil during this time period. The relationships did not appear to be affected by sex, ethnicity, income, and insurance which suggests more research is needed.

**Disclosures:**

**All Authors**: No reported disclosures

